# Development and Validation of a Multidimensional Psychometric Scale for Assessing Pain Perception and Coping Strategies Among Adolescents

**DOI:** 10.1155/prm/3924115

**Published:** 2026-06-30

**Authors:** Mengmeng Zhang, Xin Ma, Shuran Yang, Xudong Zhao, Ying Zhang, Jonas Tesarz

**Affiliations:** ^1^ Department of General Internal Medicine, Psychosomatics, and Psychotherapy, Heidelberg University, Heidelberg, Germany, heidelberg.edu; ^2^ School of Medicine, Tongji University, Shanghai, China, tongji.edu.cn; ^3^ National Clinical Research Center for Mental Disorders, Peking University Institute of Mental Health (Sixth Hospital), Beijing, China, pku.edu.cn; ^4^ Department of Psychiatry, The First Affiliated Hospital of Kunming Medical University, Kunming, China, kmmc.cn; ^5^ Department of Psychosomatic Medicine and Psychotherapy, University Medical Center, Mainz, Germany, bvdaihoc.com.vn

**Keywords:** adolescent pain, pain acceptance, pain sensitivity, psychometric validation, social–emotional impact

## Abstract

**Objectives:**

Pain is a common experience during childhood and adolescence influencing emotional well‐being, social functioning, and academic performance. Currently, there is a lack of rigorously validated multidimensional tools specifically designed for adolescents that can simultaneously assess pain sensitivity, its social–emotional impact, and pain acceptance.

**Methods:**

A sample of 836 adolescents completed the Adolescent Pain Sensitivity, Impact, and Acceptance Scale (APSIA) and the Chinese version of the Pain Sensitivity Questionnaire (PSQ) in Jiangxi Province, China (October 2023). The instrument was developed through experts’ consultations and literature review, followed by a pilot test. Exploratory factor analysis (EFA), confirmatory factor analysis (CFA), validity analyses against the PSQ, K‐means clustering, and structural equation modeling (SEM) were conducted using R.

**Results:**

The final APSIA was reduced to 18 items with three factors via EFA and CFA: pain sensitivity (8 items), social–emotional impact (6 items), and pain acceptance (4 items). The model demonstrated acceptable fit (CFI = 0.938, RMSEA = 0.072) and internal consistency (*α* > 0.80). Validity analyses against the PSQ showed statistically significant but small group differences (*p* = 0.003, *d* = 0.21) and a weak association with limited explained variance (*R* ^2^ = 0.031). Cluster analysis revealed three pain response profiles with distinct patterns in pain sensitivity, social–emotional impact, and pain acceptance. SEM revealed a strong association between pain acceptance and social–emotional impact (standardized *β* = 0.805, *p* < 0.001). Alternative directional models yielded equivalent model fit (ΔAIC < 0.001), suggesting that causal direction cannot be determined from cross‐sectional data.

**Conclusion:**

The APSIA showed initial psychometric support for assessing pain experiences in adolescents. It may provide a useful foundation for early screening, psychological intervention, and personalized pain management. Future studies should evaluate its clinical utility, cross‐cultural applicability, and long‐term stability.

## 1. Introduction

Pain is a common yet often underestimated experience in childhood and adolescence [1]. Adolescence is a critical stage characterized by significant changes both in physiological and psychological development, particularly in the maturation of pain perception and regulatory processes [2]. Chronic or recurrent pain can negatively impact adolescents’ emotional well‐being and potentially disrupt academic performance and social interactions [3, 4]. Empirical evidence suggests that adolescents with greater pain sensitivity are more susceptible to anxiety and depression, which may subsequently compromise peer interactions and diminish social support [5, 6]. Consequently, understanding adolescents’ attitudes toward pain and their coping strategies is crucial for facilitating early intervention and providing psychological support.

Pain perception is modulated by the interactions among biological, psychological, and social determinants. At the sensory level, gate control theory explains how culturally informed cognitions can “open” or “close” spinal gating, shaping perceived sensitivity [7]. High anxiety sensitivity or catastrophizing further heightens this sensitivity [8, 9]. Beyond sensation, pain behaviors themselves act as social signals: the social communication model of pain predicts that adolescents may downplay or amplify pain to protect peer standing, with attendant risks for isolation and academic disruption [10]. Recently, pain acceptance has gathered increasing attention as a key therapeutic target in psychological interventions [11]. In the adolescent developmental context, pain acceptance refers to the ability to acknowledge and tolerate pain experiences while continuing age‐appropriate academic, social, and identity‐related activities, rather than engaging in avoidance or excessive reassurance‐seeking [11]. Finally, the meaning‐making model of stress and coping frames pain acceptance as a culturally shaped reappraisal that can transform distress into growth, reducing emotional burden and fostering adaptive coping [12]. Together, these three domains—pain sensitivity, social–emotional impact, and pain acceptance—provide an integrated lens on adolescent pain [11, 13, 14]. Building on this framework, we developed the Adolescent Pain Sensitivity, Impact, and Acceptance Scale (APSIA) specifically to operationalize and quantify these three domains within a single adolescent‐appropriate instrument.

Recent advances in neuroregulation research further highlight the importance of integrating cognitive and emotional regulatory processes in pain assessment. Emerging evidence suggests that biofeedback and neurofeedback interventions may modulate pain perception, emotional regulation, and performance‐related outcomes, underscoring the multidimensional nature of pain processing [15, 16]. In addition, neurocognitive research linking working memory, anxiety regulation, and self‐regulatory mechanisms provides further support for the role of acceptance‐related processes in youth populations [17, 18]. These findings reinforce the theoretical rationale for conceptualizing adolescent pain within an integrated sensory, emotional, and acceptance‐related framework.

Emerging evidence suggests that these dimensions—pain sensitivity, social–emotional impact, and pain acceptance—are not independent but dynamically interconnected. For instance, adolescents with heightened pain sensitivity may be more vulnerable to emotional disturbances and social challenges, especially when they lack adequate coping resources or social support [19, 20]. Conversely, higher levels of pain acceptance have been linked to reduced emotional reactivity and improved interpersonal functioning, which may potentially mitigate the negative effects of pain sensitivity [5, 21]. Although these dimensions are conceptually distinguishable and closely related, their interactions may significantly influence adolescents’ overall pain experience, affecting both psychological well‐being and functional outcomes. Therefore, understanding the interactions between these domains is essential for developing effective, personalized pain interventions in adolescence.

Despite increasing recognition of the multidimensional nature of pain, current assessment tools often focus narrowly on isolated components. For instance, the Pain Sensitivity Questionnaire (PSQ) focuses on sensory experiences, the Pain Catastrophizing Scale (PCS) assesses maladaptive cognitive patterns, and the Chronic Pain Anxiety Symptoms Scale (CPASS) emphasizes fear‐avoidance behavior [22, 23]. The Pain Coping Questionnaire (PCQ) and the Survey of Pain Attitudes (SOPA) examine pain coping strategies and attitudes; however, their measurement dimensions remain constrained [24–26]. Moreover, these instruments fail to fully integrate the social–emotional impact of pain or the adaptive role of pain acceptance in adolescents. In addition, many of them were developed for adult individuals and may lack developmental appropriateness for younger populations. This highlights a critical gap in reliable and valid tools capable of assessing pain sensitivity, social–emotional impact, and pain acceptance in a unified framework. To address this gap, the present study aims to develop and validate the APSIA, a multidimensional psychometric instrument specifically designed for adolescents.

Based on these theoretical frameworks, we formulated the following a priori hypotheses (H).

 H1: Adolescent pain experiences would be best represented by a multidimensional structure comprising pain sensitivity, social–emotional impact, and pain acceptance.
 H2: These three dimensions would demonstrate significant statistical associations, reflecting dynamic interconnections among sensory, social–emotional, and cognitive acceptance processes.
 H3: The multidimensional structure would demonstrate acceptable psychometric properties, including internal consistency, construct validity, and measurement invariance across gender and age groups.

## 2. Methods

### 2.1. Participants

In October 2023, this study enrolled school‐aged children and adolescent participants from 22 schools across Jiangxi Province, China. Recruitment procedures were conducted with approval and cooperation of school administrators and teachers. Both participants and their parents were fully informed about the study’s objectives, voluntary nature, and confidentiality measures. Participants were included if they were school‐aged children or adolescents younger than 18 years and capable of independently completing the questionnaire. This age range enabled scale calibration across developmental stages spanning late childhood, early adolescence (< 13 years), middle adolescence (14–16 years), and late adolescence (17‐18 years). Individuals with cognitive impairments or preexisting psychiatric conditions were excluded to reduce confounding variables that might affect pain perception and emotional processing. This exclusion criterion was implemented to ensure a relatively homogeneous nonclinical sample for initial scale development. This research adhered to the principles outlined in the Declaration of Helsinki, and ethical approval was granted by the Ethics Committee of Tongji University (Approval No. 2021tjdx062). Both participants and their parents were fully informed about the study objectives, voluntary nature, and confidentiality procedures, and written informed consent was obtained from both participants and their legal guardians.

### 2.2. Instrument Development

The APSIA was developed to evaluate adolescent pain sensitivity, social–emotional impact, and pain acceptance (Appendix A, Supporting Information). Its development was based on an extensive literature review and incorporated insights from established pain‐related measures such as the McGill Pain Questionnaire (MPQ), PCS, and Pain Attitudes Questionnaire (PAQ) [27–29]. Then, a panel of four experts in adolescent psychology and pain research (≥ 10 years clinical research experience) analyzed and discussed these instruments, leading to the construction of items tailored to adolescents. Each expert independently rated every draft item on a 4‐point relevance scale (1 = not relevant to 4 = highly relevant). Items with an item‐content‐validity index (I‐CVI) ≥ 0.78 were retained; those below the threshold were revised or deleted after a consensus meeting.

The initial APSIA framework comprised 33 items, reflecting multiple dimensions of pain experiences and responses, including (1) anticipation and awareness of pain (e.g., “I easily feel pain”), (2) social–emotional and functional consequences (e.g., “When I feel pain, I tend to withdraw from social interactions”), and (3) coping and acceptance strategies (e.g., “I use relaxation techniques to reduce my pain experience”). Each item was measured using a 5‐point Likert scale ranging from 1 (strongly disagree) to 5 (strongly agree), with certain items reverse‐coded to mitigate response bias.

A pilot study was conducted to evaluate the questionnaire’s clarity and applicability through a small‐scale pretest with 20 adolescents (11 girls, 9 boys; mean age = 13.4 ± 1.2 years). Thirteen were in Grade 7 and seven in Grade 8. All attended public schools in Jiangxi Province. Participants were community‐dwelling adolescents recruited through school notices. All participants provided feedback regarding the clarity of language and the relevance of the items. Then, items identified as redundant, ambiguous, or exhibiting low discriminatory power were either removed or revised. For instance, the item “I am extremely sensitive to small pains” was eliminated due to its conceptual overlap with “I often find mild pain unbearable.” The item “I feel more secure when carrying painkillers” was discarded as it elicited uniform responses from participants, indicating a lack of variability. Additionally, “I need a longer time to adjust to pain intensity” was rephrased to “Small injuries cause prolonged pain for me” for clarity, as participants encountered comprehension difficulties. After these modifications, the revised APSIA consisted of 29 items across three dimensions. The final questionnaire and all comparator measures were administered in Simplified Chinese.

### 2.3. Data Collection Procedure

The survey was distributed via online platforms and took approximately 15–20 min to complete. Each class moved to the school computer laboratory, where desktops displayed a landing page with a URL linking to the Wenjuanxing survey. To ensure standardized data collection procedures, a well‐trained research team comprising two postgraduate students and one undergraduate student was present in every lab to deliver a brief scripted introduction, offer technical help, and answer nonleading questions. Parental written consent had been obtained 1 week earlier via paper forms distributed by teachers; adolescents provided electronic assent on the first survey page. The platform automatically logged completion time (median = 17 min, interquartile range [IQR] = 15–20 min), enforced CAPTCHA verification to reduce automated responses, and blocked repeat submissions from the same IP. All instruments were administered in Simplified Chinese, and responses were anonymized before analysis.

The study was conducted across 22 schools in Jiangxi Province using standardized data collection procedures. Each questionnaire included clear instructions to assist participants in understanding and accurately responding to the items, and all data were anonymized to maintain confidentiality and uphold ethical standards.

To enhance data reliability and minimize response bias, several control measures were implemented. The items of the APSIA were presented in a randomized order to minimize order effects, and attention check questions were embedded throughout the questionnaire to identify inattentive respondents. Responses that were incomplete, exhibited excessive missing data, or failed attention checks were excluded from subsequent analysis.

As part of the validity assessment, the PSQ was administered alongside the APSIA. The PSQ comprises 17 items, each describing an everyday scenario (e.g., “taking a warm shower” and “getting a splinter”). Respondents rate the imagined pain intensity on an 11‐point numeric scale (0 = “no pain” to 10 = “worst pain imaginable”). The PSQ targets sensory pain sensitivity, serving as a complementary construct for convergent validity. A professionally translated and validated Chinese version of the PSQ was utilized in this study [30, 31]. Participants completed the PSQ immediately after the APSIA, allowing for direct comparisons in pain sensitivity measurements.

### 2.4. Statistical Analysis

Statistical analyses were conducted using R 4.2.1. In R, the *psych* package was used for exploratory factor analyses (EFAs), *lavaan* package was used for confirmatory factor analyses (CFAs), *mclust* was used for cluster and latent class analyses, and *semPlot* was used for path analysis in structural equation modeling (SEM) [32, 33]. The analyses included EFA, CFA, cluster analysis, and path analysis to evaluate the questionnaire’s psychometric properties [33, 34]. Analysis of variance (ANOVA) was conducted to examine group differences between predefined pain‐sensitivity groups.

To identify the factor structure, an EFA was performed using the minimum residual extraction (minres) method with oblique (oblimin) rotation. Items were evaluated based on factor loadings (excluding those below 0.50), communalities (removing those under 0.40), and complexity (prioritizing deletion for values above 2.0). CFA was subsequently conducted to validate the factor structure, using fit indices like comparative fit index (CFI), Tucker–Lewis index (TLI), and root mean square error of approximation (RMSEA) [35, 36]. Items with low loadings, high residuals, or redundancy were removed to improve model fit and clarity.

Participants were categorized into high and low pain sensitivity groups based on PSQ score to explore differences in pain sensitivity and attitudes. The APSIA’s known‐group validity and concurrent validity were evaluated. K‐means and hierarchical clustering were applied to analyze item classification patterns, aiding questionnaire refinement by identifying distinct item groupings related to pain perception and coping [37]. Path analysis and SEM were used to examine relationships among latent variables. Statistical tests used a significance threshold of *p* < 0.05, with model refinements based on empirical fit indices for robustness and clarity.

## 3. Results

### 3.1. Descriptive Statistics of Items and Initial Screening

**FIGURE 1:**
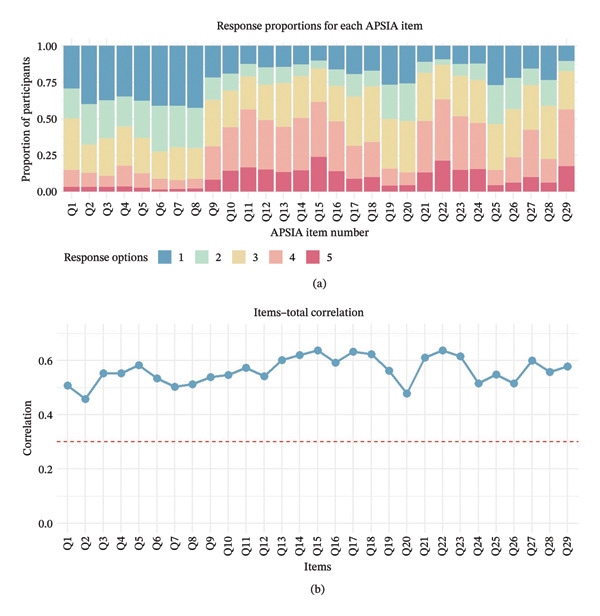
Item score distributions and item–total correlations. (a) The fitted score frequencies for all questionnaire items, categorized by response levels ranging from 1 to 5 (strongly disagree to strongly agree). Each line represents the smoothed trend of response distributions across the items. (b) The item–total correlations for all questionnaire items, indicating the strength of the relationship between each item and the overall scale score. The dashed red line denotes the commonly accepted threshold (*r* = 0.30) for acceptable item discrimination.

A total of 836 participants were included in the analysis. Participant characteristics are detailed in Appendix B (Supporting Information). The initial version of APSIA comprised 29 items distributed across four dimensions including pain sensitivity, social impact, acceptance, and emotional impact. Descriptive statistics, presented in Appendix C (Supporting Information), show mean scores ranging from 1.97 to 3.62 and standard deviations between 1.01 and 1.32, indicating moderate variability across the measured constructs. Skewness values ranged from −0.89 to 0.84, with negative skewness observed in items related to pain normalization and coping (e.g., “I want others to understand and respect my pain,” −0.89), indicating a tendency for participants to endorse higher scores on these items. Conversely, positive skewness was observed in items related to pain sensitivity (e.g., “My pain experiences are more frequent than most people,” 0.84), indicating that the majority of participants provided lower ratings, with fewer individuals selecting higher scores. Kurtosis values were predominantly negative, indicating a relatively flat distribution. Scores for items assessing pain sensitivity and social impact were distributed across all score levels, while items related to acceptance were concentrated at the higher end of the scale (Figure 1). Item–total correlations, as depicted in Figure 1(a), ranged from 0.42 to 0.62, surpassing the 0.30 threshold.

### 3.2. EFA and CFA Findings

An EFA using minimum residual estimation and oblique rotation was conducted, applying exclusion criteria of factor loadings below 0.50 or complexity exceeding 2.0 (e.g., Q9–Q11). Parallel analysis suggested the retention of up to four factors. However, the fourth factor demonstrated weak and conceptually diffuse loadings and did not form interpretable domains. In contrast, the three‐factor solution yielded clear and theoretically coherent factor structures. The retained three‐factor solution provided the most parsimonious and theoretically interpretable structure and was therefore carried forward to CFA and subsequent analyses. The first factor, pain sensitivity, included eight items such as “I feel stronger pain when injured,” with a loading of 0.857, explaining 29% of the total variance. The second factor, social–emotional impact, contained six items such as “I want others to understand and respect my pain,” with a loading of 0.785, accounting for 22% of the variance. The third factor, pain acceptance, had four items, with “Accepting pain is part of personal growth” showing a loading of 0.818, contributing 10% of the variance. Together, these three factors explained 61% of the total variance. The Kaiser–Meyer–Olkin (KMO) measure of sampling adequacy was 0.936, indicating acceptable suitability for factor analysis. Bartlett’s test of sphericity was significant [*χ* ^2^ (406) = 14094.25, *p* < 0.001], confirming that the correlation matrix was appropriate for factor extraction. Harman’s single‐factor test indicated that the first unrotated factor accounted for 32.02% of the variance, below the commonly referenced 40% threshold, suggesting no severe common method bias.

The result of CFA supported the structural validity of the model, as shown in Table 1 and Appendix D (Supporting Information). Items Q12, Q13, Q25, and Q26 were removed due to high residual variance and low factor specificity, suggesting that these items introduced measurement error and did not align well with the latent constructs. Further exclusions of Q18, Q19, Q20, and Q28 were based on low factor loadings and high cross‐loadings, indicating that these items either contributed weakly to their designated factors or demonstrated substantial overlap with multiple constructs. The final confirmatory model demonstrated acceptable model fit (CFI = 0.938, TLI = 0.923, RMSEA = 0.072), with all standardized factor loadings ranging between 0.645 and 0.857. Correlations among the three factors were high (0.87–0.97), indicating substantial shared variance across APSIA dimensions. Variance inflation factors (VIFs < 5) and discriminant validity indices did not indicate problematic multicollinearity. Convergent validity was supported by average variance extracted (AVE = 0.529–0.643) and composite reliability (CR = 0.843–0.935), all exceeding recommended thresholds. In addition, multigroup CFA was conducted to examine measurement invariance across both gender and age groups. For gender (female = 433, male = 403), configural, metric, and scalar invariances were supported (metric: ΔCFI = −0.001, ΔRMSEA = +0.001; scalar: ΔCFI < 0.001, ΔRMSEA = +0.001), meeting recommended criteria (|ΔCFI| ≤ 0.01; |ΔRMSEA| ≤ 0.015). For age groups (< 14 vs. ≥ 14 years), configural, metric, and scalar invariance models were tested sequentially. Fit remained stable across increasingly constrained models (configural: CFI = 0.993, RMSEA = 0.079; metric: ΔCFI = −0.001, ΔRMSEA = +0.002; scalar: ΔCFI < 0.001, ΔRMSEA = +0.001), meeting recommended criteria (|ΔCFI| ≤ 0.01; |ΔRMSEA| ≤ 0.015), supporting scalar invariance across age groups.

**TABLE 1:** Factor loadings from confirmatory factor loadings.

Question	Estimate	Std. err	*z* value	*p* (>|*z*|)	Std. all
I easily feel pain.	1.000	—	—	—	0.723
Small pain is hard to bear.	1.027	0.049	21.057	< 0.001	0.738
I am more sensitive to pain than others my age.	1.153	0.048	24.171	< 0.001	0.843
Anxiety increases my sensitivity to pain.	1.167	0.051	22.682	< 0.001	0.793
I feel stronger pain than other people when injured.	1.185	0.048	24.597	< 0.001	0.857
Small injuries cause prolonged pain for me.	1.049	0.044	23.991	< 0.001	0.837
I feel pain more easily due to fatigue than others.	1.040	0.044	23.490	< 0.001	0.820
My pain experiences are more frequent than most people.	1.034	0.045	22.800	< 0.001	0.797
Pain is a reminder to slow down.	1.000	—	—	—	0.763
I want others to understand and respect my pain.	1.031	0.045	22.978	< 0.001	0.785
Pain makes one stronger.	1.110	0.047	23.646	< 0.001	0.806
My friends and family often ask about my pain.	0.909	0.048	18.777	< 0.001	0.654
I have friends at school with whom I share pain.	0.895	0.048	18.494	< 0.001	0.645
I forget pain more easily when I am with friends.	0.932	0.047	19.993	< 0.001	0.693
Accepting pain is part of personal growth.	1.000	—	—	—	0.818
I often expect pain to disappear quickly.	0.793	0.041	19.175	< 0.001	0.648
Pain is a normal part of everyday life.	1.004	0.043	23.588	< 0.001	0.770
Pain helps us understand ourselves better.	0.978	0.041	24.110	< 0.001	0.785

### 3.3. Validity Assessment Against the PSQ

**FIGURE 2:**
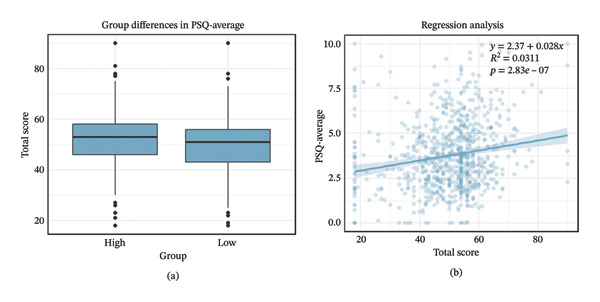
Split‐half reliability and discriminant validity. (a) presents a discriminant validity test utilizing Pain Sensitivity Questionnaire (PSQ) scores. Participants were categorized into High‐PS Group and Low‐PS Group based on median splits. The box plots illustrate the total scores of the newly developed scale for each group, revealing a statistically significant difference between the two groups. (b) depicts a predictive validity analysis conducted through linear regression. The total score of the APSIA served as a predictor for the average PSQ score. The regression model demonstrated a significantly positive relationship (*p* < 0.001), although the explained variance was relatively modest (*R* ^2^ = 0.031).

To assess the known‐group validity of the pilot version of APSIA, participants were categorized into high pain sensitivity group (High‐PS Group, *n* = 417) and low pain sensitivity group (Low‐PS Group, *n* = 419) based on the median total score of PSQ. This median split was used as an exploratory grouping strategy to compare APSIA scores across relatively higher and lower pain sensitivity levels in the absence of established PSQ clinical cutoffs for adolescents. An independent samples *t*‐test revealed significantly higher scores in the High‐PS Group (mean = 51.30, SD = 12.20) compared to the Low‐PS Group (mean = 48.79, SD = 12.00, *t* = 2.999, *p* = 0.003, 95% CI [0.868, 4.157]). However, the group difference was small in magnitude (Cohen’s *d* = 0.21), indicating limited practical separation between the PSQ‐defined groups (Figure 2(a)).

To evaluate concurrent validity against the PSQ, a linear regression analysis was conducted, with the APSIA total score as the predictor and the PSQ average score as the outcome. The regression model showed a statistically significant positive association (*β* = 0.028, SE = 0.0054, *t* = 5.177, *p* < 0.0001). However, the explained variance was small (*R* ^2^ = 0.031, Figure 2(b)), indicating that the APSIA accounted for only 3.1% of the variance in PSQ scores. Overall, these findings indicate a statistically significant but weak association between APSIA and PSQ scores.

### 3.4. Age and Gender Differences

Independent samples *t*‐tests were conducted to examine whether APSIA factor scores differed by age group (< 14 vs. ≥ 14 years) and by gender (male vs. female). Means, *t*‐statistics, 95% confidence intervals, and standardized effect sizes (Cohen’s *d*) are summarized in Table 2. Older adolescents scored significantly lower on the social–emotional impact factor (Factor 2), *d* = 0.222, *p* = 0.003, indicating a small reduction in pain‐related social–emotional impact with age. Females reported slightly higher pain sensitivity (Factor 1) than males, *d* = −0.214, *p* = 0.002. No significant age or gender differences were observed for the pain acceptance factor (Factor 3), and remaining effects were negligible (|*d*| < 0.12).

**TABLE 2:** Group differences in APSIA factor scores by age and gender.

Factor	Group	< 14/male	≥ 14/female	*t*	*d* [95% CI]	*p*
Factor 1	Age < 14 vs. ≥ 14	2.11	2.15	−0.55	−0.041 [−0.191, 0.109]	0.584
Factor 2	Age < 14 vs. ≥ 14	3.41	3.21	2.95	0.222 [0.071, 0.372]	0.003
Factor 3	Age < 14 vs. ≥ 14	3.26	3.19	0.85	0.066 [−0.084, 0.216]	0.395
Factor 1	Gender M vs. F	2.02	2.22	−3.1	−0.214 [−0.350, −0.078]	0.002
Factor 2	Gender M vs. F	3.33	3.37	−0.6	−0.042 [−0.178, 0.094]	0.547
Factor 3	Gender M vs. F	3.3	3.18	1.62	0.113 [−0.023, 0.248]	0.106

### 3.5. Cluster Analysis

Internal clustering indices yielded mixed results: the silhouette index and mclust favored a two‐cluster solution (0.413 vs. 0.372), whereas the Calinski–Harabasz index favored three clusters (522.59 vs. 439.99). Bootstrap stability analysis indicated greater robustness for the three‐cluster solution (mean ARI = 0.830, SD = 0.113) compared to the two‐cluster solution (mean ARI = 0.631, SD = 0.368).

**FIGURE 3:**
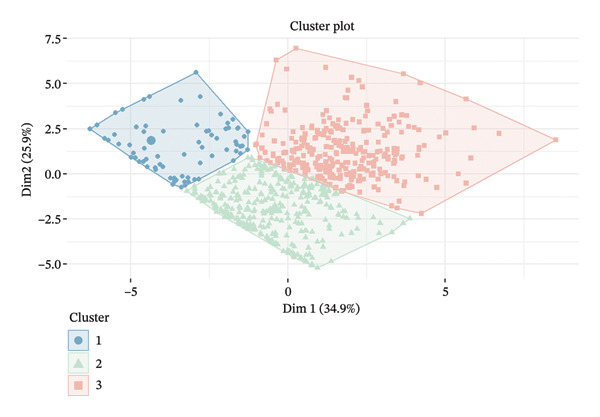
Cluster analysis results. The scatter plot illustrates the clustering results based on K‐means clustering. Three distinct clusters were identified and are represented in different colors and shapes: Cluster 1 (red, circles): individuals with low pain sensitivity; Cluster 2 (green, triangles): individuals with moderate pain sensitivity and high social support needs; and Cluster 3 (blue, squares): individuals with high pain sensitivity. The axes represent the two principal dimensions extracted through PCA, explaining 34.9% (Dim1) and 25.9% (Dim2) of the variance, capturing the main variance in the dataset.

K‐means clustering analysis identified three distinct participant clusters: Cluster 1 (“detached minimizers,” *n*_1_ = 114), Cluster 2 (“high‐impact acceptors,” *n*_2_ = 367), and Cluster 3 (“vulnerable internalizers,” *n*_3_ = 355) (see Appendix E [Supporting Information] and Figure 3).

Detached minimizers exhibited the lowest scores in pain sensitivity (1.39 ± 0.14), social–emotional impact (1.74 ± 0.20), and pain acceptance (1.57 ± 0.21). Participants in this cluster showed a pain response pattern characterized by reduced pain perception, lower pain acceptance, and limited pain‐related social interactions. High‐impact acceptors exhibited a distinct profile: with moderate pain sensitivity (1.59 ± 0.15) but the highest social–emotional impact (3.79 ± 0.23), indicating greater emotional and social responses to pain. Vulnerable internalizers included participants with the highest pain sensitivity (2.99 ± 0.16) but only moderate social–emotional impact (3.43 ± 0.14) and pain acceptance (3.36 ± 0.29, see Appendix E [Supporting Information]).

### 3.6. Path Analysis

**FIGURE 4:**
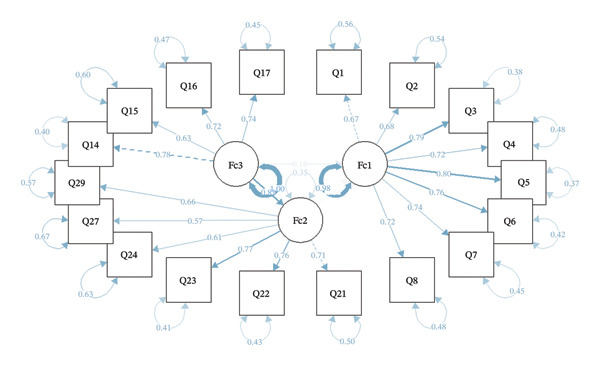
Structural equation model for path analysis. The diagram illustrates the SEM employed in the path analysis, comprising three latent constructs: Fc1 (pain sensitivity), assessed through items Q1 to Q8; Fc2 (social and emotional impact), evaluated via items Q21 to Q29; and Fc3 (acceptance and meaning), measured by items Q14 to Q17. The directional arrows depict the hypothesized relationships among these constructs. The thickness of the arrows signifies the strength of these relationships, with numerical values representing standardized path coefficients. Curved arrows indicate correlations between observed variables, while dashed lines denote weaker or nonsignificant relationships.

The SEM was employed to examine the proposed relationships among three latent constructs: Factor 1 (pain sensitivity), Factor 2 (social–emotional impact), and Factor 3 (pain acceptance), as detailed in Appendix F (Supporting Information) and Figure 4. Factor 1 was measured by eight observed variables (Q1–Q8), Factor 2 by six (Q21–Q29), and Factor 3 by four (Q14–Q17). The structural model specified directional paths among the three latent factors for analytical purposes. Although the chi‐square test was significant (*χ* ^2^ (132) = 644.705, *p* < 0.001), which is expected due to its sensitivity to large sample size, alternative fit indices were also considered. Alternative model fit indices indicated an acceptable model fit: CFI = 0.927, TLI = 0.915, RMSEA = 0.068 (90% CI: 0.063–0.073), and SRMR = 0.051.

All factor loadings in the measurement model were statistically significant (*p* < 0.001), ranging from 0.573 to 0.796. In the structural model, Factor 3 was strongly associated with Factor 2 (*β* = 0.710, standardized *β* = 0.805, *p* < 0.001). VIFs (all < 5, maximum ≈ 4.27) and HTMT ratios (< 0.85) did not indicate problematic multicollinearity. To examine the robustness of the structural specification, an alternative reversed model (Factor 2 ⟶ Factor 3) was tested and demonstrated virtually identical model fit (ΔAIC < 0.001; ΔBIC < 0.001). The reversed model yielded virtually identical fit indices (CFI = 0.938, TLI = 0.928, RMSEA = 0.072). The path from Factor 3 to Factor 1 was marginally significant at the 0.1 level (*β* = 0.126, standardized *β* = 0.160, *p* = 0.062). Conversely, Factor 2 did not significantly predict Factor 1 (*β* = −0.035, standardized *β* = −0.040, *p* = 0.640). The residual variances for Factor 1 and Factor 2 were 0.228 and 0.103, respectively (*p* < 0.001). For model identification purposes, the variance of Factor 3 was fixed at 1.000. The error variances for all observed variables were significant (*p* < 0.001), with standardized values ranging from 0.366 to 0.672, indicating moderate measurement error.

## 4. Discussion

This research developed and preliminarily validated the APSIA for adolescents, aiming to address gaps in current pain assessment instruments related to the social–emotional impact and acceptance of pain (Appendices A, F, and G, Supporting Information). Factor analysis provided initial support for a three‐factor structure encompassing pain sensitivity, social–emotional impact, and pain acceptance. The findings also indicate that pain acceptance can be measured in adolescence, although further research is needed to clarify its relation to distress and coping. Because the scale was calibrated in a school‐based cohort with very low chronic pain prevalence, these factors represent normative benchmarks rather than disease‐specific profiles.

From a theoretical standpoint, the findings are consistent with the biopsychosocial model of pain, reflecting the relevance of cognitive, emotional, and social determinants of adolescent pain [38, 39]. Besides, they also resonate with the gate control account of sensory modulation, the social‐communication view of relational pain display, and the meaning‐making perspective on adaptive appraisal, thereby situating the results firmly within a biopsychosocial framework [7, 10, 12]. These findings support further examination of peer and family support, as well as cognitive regulation processes, in adolescent pain assessment and management [40].

By validating a three‐factor structure encompassing pain sensitivity, social–emotional impact, and pain acceptance, this study provided preliminary evidence that these dimensions are distinguishable but strongly related aspects of adolescent pain experience. Although the three APSIA dimensions were highly correlated, they were retained as separate but closely related constructs because they represent different aspects of adolescent pain experience: pain sensitivity reflects sensory reactivity to pain, social–emotional impact reflects the emotional and interpersonal consequences of pain, and pain acceptance reflects acceptance‐related appraisal and coping. Importantly, maintaining this distinction may also be clinically meaningful because the three dimensions point to partially different intervention targets. Elevated pain sensitivity may indicate the relevance of interventions addressing pain‐related threat learning, central sensitization, and altered sensorimotor processing, such as pain neuroscience education, graded exposure, or sensorimotor retraining [41, 42]. In contrast, elevated social–emotional impact may highlight the need for approaches targeting emotional processing, interpersonal functioning, and trauma‐ or stress‐related contributors to pain where clinically indicated, such as emotional awareness and expression therapy or interpersonal and trauma‐focused interventions [43, 44]. Finally, lower pain acceptance may point to acceptance‐ and coping‐based approaches, including acceptance and commitment therapy or mindfulness‐based interventions [45, 46]. Thus, although the APSIA dimensions are strongly interrelated, distinguishing them may help generate more specific hypotheses about intervention needs and mechanisms in future clinical research. Nevertheless, this distinction should be interpreted cautiously given the high correlations among the three dimensions. At the same time, the strong correlations may indicate the presence of a broader pain‐related construct, which warrants further examination in future studies. For instance, cluster analysis grouped participants by their APSIA scores into profiles with different combinations of sensitivity, social–emotional impact, and acceptance; the clinical meaning of these profiles awaits replication with external distress or coping measures [47]. The statistically significant association with the PSQ should not be overinterpreted, as the known‐group effect size was small (Cohen’s *d* = 0.21) and APSIA explained only 3.1% of the variance in PSQ scores. Path analysis revealed a strong statistical association between pain acceptance and social–emotional impact. However, alternative directional models demonstrated equivalent statistical fit, suggesting that the relationship may reflect conceptual proximity rather than a unidirectional causal pathway. These observations suggest that acceptance‐related processes merit further investigation in adolescent pain research. Notably, the lack of a direct link from social–emotional difficulties to pain sensitivity suggests that affective experiences alone may not dictate how adolescents perceive pain physically.

From an applied perspective, recent evidence indicates that neurofeedback‐based and biofeedback‐based interventions may be relevant to emotional regulation and chronic pain management, suggesting potential avenues for tailored intervention strategies [15, 16]. The present multidimensional framework may therefore inform stratified screening approaches and regulation‐focused intervention programs for adolescents with differing pain profiles. Compared to existing tools that often isolate pain sensitivity, negative thoughts, or fear‐avoidance behaviors, the APSIA may provide a broader assessment by incorporating three interrelated domains [48]. For instance, while the PSQ captures sensory reactivity and the PCS evaluates maladaptive cognitive patterns, these instruments do not assess social functioning or adaptive acceptance strategies—two critical aspects of adolescent pain coping [27, 49]. Previous experimental studies have similarly reported that both gender and prior competitive or agonistic experiences may influence perceived pain responses, highlighting the importance of considering individual differences in pain perception research [50]. Additionally, tools such as the PAQ and the CPASS target adult populations or focus narrowly on fear responses [22, 23]. By capturing both pain‐related distress and adaptive meaning‐making, the APSIA offers a developmental perspective suited for the adolescent context.

In educational settings, the APSIA may have practical value as a research and screening tool, pending further validation. Normative school data can help flag students with extreme scores, although psychometric properties still need replication in clinic samples. School‐based applications could include using the APSIA in research or preliminary screening contexts to identify adolescents with elevated pain‐related distress. Once cluster validity is confirmed against behavioral outcomes, interventions could be tailored to each profile [51].

Although the APSIA has demonstrated validity evidence in adolescent populations, it is important to recognize several methodological limitations. First, the cross‐sectional study design precludes causal inferences. While relationships among pain sensitivity, social–emotional impact, and pain acceptance were modeled, the directionality of these effects remains uncertain. Second, the study’s reliance on self‐reported questionnaires introduces the potential for cognitive biases and social desirability effects. Accordingly, future studies may consider complementing self‐report with behavioral or observational indicators. Furthermore, the APSIA was validated solely using subjective self‐report data and was not compared with objective or psychophysiological indicators of pain. Third, although the PSQ was used as a validated comparator, it mainly captures sensory pain sensitivity. Therefore, the lack of external measures for social–emotional impact and pain acceptance limited the external validation of the APSIA. Fourth, although measurement invariance across both gender and age groups was supported, test–retest reliability and responsiveness to change were not evaluated in the present study. Fifth, cross‐validation using independent samples was not conducted, and replication in separate cohorts would strengthen generalizability. Sixth, although discriminant validity indices (e.g., HTMT) did not indicate severe redundancy, the high inter‐factor correlations suggest meaningful conceptual overlap among the three APSIA dimensions. A broader general pain‐experience factor may partly account for the observed associations, and future studies should formally compare correlated‐factor, higher‐order, and bifactor models in independent samples. Seventh, the pilot study (*N* = 20) provided preliminary feedback on item clarity; however, its limited sample size and the absence of formal cognitive interviewing procedures restrict the depth of qualitative validation. Future research should incorporate structured qualitative methods to further refine item interpretation. Furthermore, the primary validation of the APSIA was conducted within a community adolescent cohort, resulting in limited representation of individuals experiencing chronic pain. Moreover, item wording was refined without formal cognitive interviewing; future pretest strategies would strengthen item optimization. In addition, the exclusion of adolescents with diagnosed psychiatric disorders limits the applicability of the findings to clinical populations. Future validation studies should include diverse clinical samples to examine external validity. Because the sample consisted exclusively of adolescents from China, cultural differences in pain expression and coping may limit the international generalizability of the findings. Finally, the sample lacked adolescents with persistent pain, so items specific to that group (e.g., carrying analgesics for reassurance) may be under‐represented; validating the APSIA in chronic pain samples and revisiting such items are therefore essential.

Future research should include adolescents with chronic pain and other clinical groups to evaluate the APSIA across more diverse contexts. Further studies should also examine its cross‐cultural applicability, test–retest reliability, responsiveness to change, and associations with longitudinal mental health and functional outcomes.

## 5. Conclusion

This study developed and preliminarily validated the APSIA as a measure of adolescent pain experience across three closely related dimensions: pain sensitivity, social–emotional impact, and pain acceptance. Psychometric analyses provided initial support for its three‐factor structure, acceptable reliability, and modest external validity evidence against the PSQ. However, the high inter‐factor correlations and small APSIA–PSQ association indicate that further validation is needed, particularly through independent replication, additional external criteria, and testing in adolescents with chronic or recurrent pain.

## Author Contributions

Mengmeng Zhang: conceptualization, methodology, software, validation, formal analysis, data curation, writing–original draft, and writing–review and editing. Xin Ma: formal analysis, validation, visualization, and writing–review and editing. Shuran Yang: investigation and project administration. Xudong Zhao: investigation and resources. Ying Zhang: supervision, methodology, and writing–review and editing. Jonas Tesarz: conceptualization, supervision, funding acquisition, and writing–review and editing. All authors contributed substantially to the study.

## Funding

This work was supported by the German Research Foundation, SFB1158; German Federal Ministry of Education and Research, 01EC1904A; and China Scholarship Council, 10.13039/501100004543, 202306260043. Open Access funding enabled and organized by Projekt DEAL.

## Disclosure

Patients or members of the public were not involved in the design, conduct, reporting, or dissemination plans of this research. All authors approved the final manuscript and agree to be accountable for all aspects of the work. The funding bodies had no role in the study design, data collection, analysis, interpretation, or manuscript preparation.

## Ethics Statement

This study was approved by the Ethics Committee of Tongji University (Approval No. 2021tjdx062). Written informed consent was obtained from all participants and their legal guardians prior to participation. All procedures adhered to the principles of the Declaration of Helsinki.

## Conflicts of Interest

The authors declare no conflicts of interest.

## Supporting Information

Additional supporting information can be found online in the Supporting Information section.

## Supporting information


**Supporting Information** Supporting Information accompanying this article includes the following. Appendix A: Study flowchart. Appendix B: Sample characteristics. Appendix C: Descriptive statistics of APSIA items. Appendix D: Original and refined factor structures. Appendix E: Cluster‐specific item means. Appendix F: Standardized factor loadings and SEM coefficients. Appendix G: Sample survey items.

## Data Availability

The dataset generated and analyzed during the current study is not publicly available due to participant confidentiality and ethical restrictions. However, anonymized data may be made available from the corresponding author upon reasonable request and with permission from the relevant institutional ethics board.
