# Supplementary material for: Development and Validation of a Multidimensional Psychometric Scale for Assessing Pain Perception and Coping Strategies Among Adolescents
Source: Pain Res Manag. 2026 Jun 30;2026:3924115. doi: 10.1155/prm/3924115 (PMC13318478; doi:10.1155/prm/3924115)
Supplement: Supplementary file 1 — Supporting Information Supporting Information accompanying this article includes the following. Appendix A: Study flowchart. Appendix B: Sample characteristics. Appendix C: Descriptive statistics of APSIA items. Appendix D: Original and refined factor structures. Appendix E: Cluster‐specific item means. Appendix F: Standardized factor loadings and SEM coefficients. Appendix G: Sample survey items. [file PRM-2026-3924115-s001.docx]

**Supplemental Material**

Appendix A: Flowchart

Appendix B: Sample Characteristics

Appendix C: Descriptive statistics for responses to the Pain Sensitivity and Attitude Questionnaire (APSIA)

Appendix D: The Original and Refined Scale Structures

Appendix E: Mean Scores for Each Cluster Across Questionnaire Items

Appendix F: Standardized Factor Loadings and Path Coefficients in the Structural Equation Model

Appendix G: Sample Survey Items

**Appendix A: Flowchart**


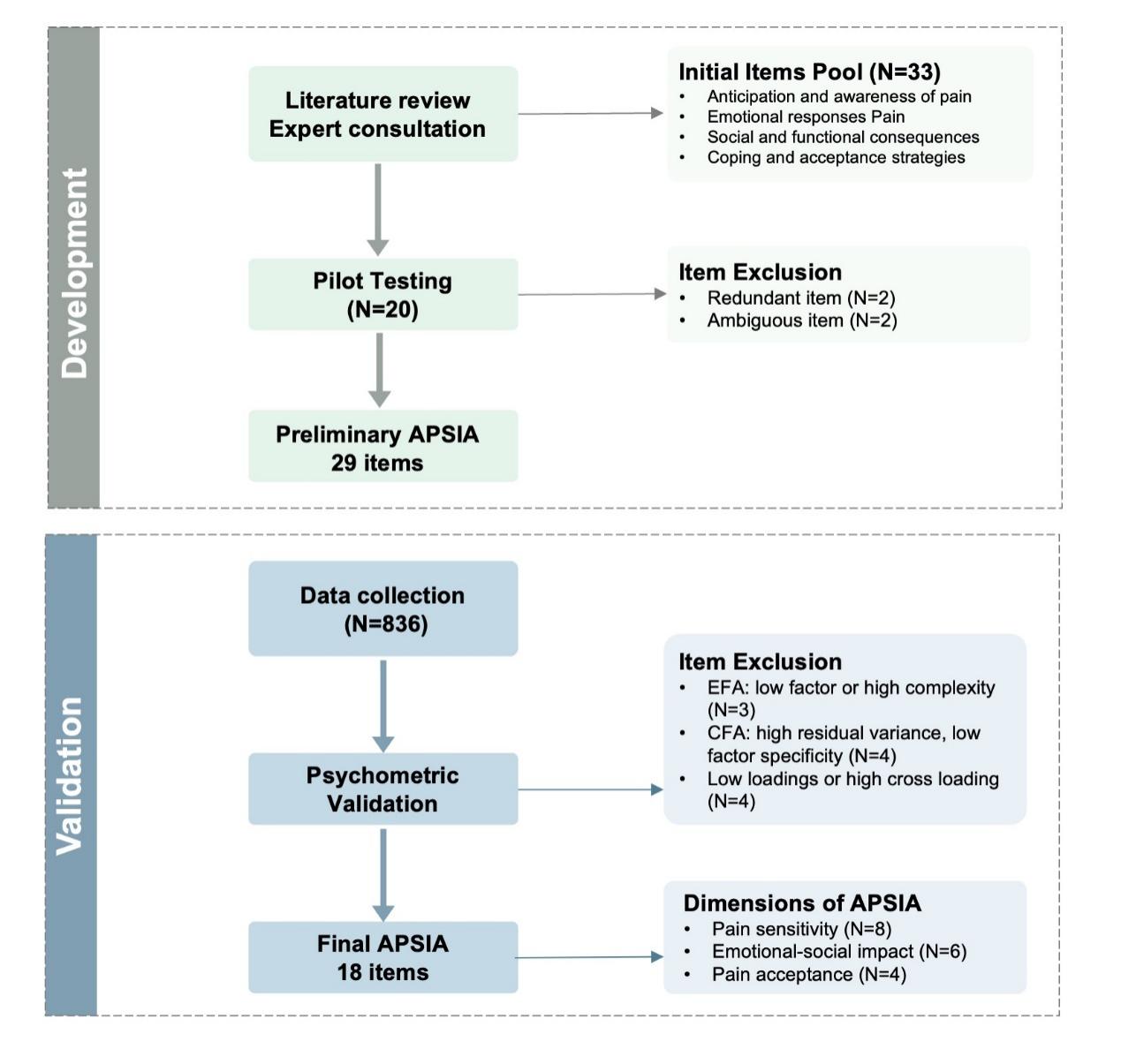


**Appendix B:**

| **Appendix B: Sample Characteristics** | | |
| --- | --- | --- |
| Variable | Category | Mean(SD)/N (%) |
| Gender | Male | 403 (48.21%) |
|  | Female | 433 (51.79%) |
| Grade | | 7.0 (2.5) |
| Age | | 11.8 (3.0) |
| One Child | Yes | 690 (82.54%) |
|  | No | 146 (17.46%) |
| Area | City | 501 (59.93%) |
|  | Rural | 335 (40.07%) |

**Appendix C:**

| **Appendix C: Descriptive statistics for responses to the Pain Sensitivity and Attitude Questionnaire (APSIA)** | | | | | | | |
| --- | --- | --- | --- | --- | --- | --- | --- |
| **Item** | **Question** | **Mean** | **SD** | **Median** | **Skewness** | **Kurtosis** | **SE** |
| Q1 | I easily feel pain. | 2.39 | 1.11 | 3 | 0.24 | -0.81 | 0.04 |
| Q2 | Small pain is hard to bear. | 2.08 | 1.12 | 2 | 0.78 | -0.31 | 0.04 |
| Q3 | I am more sensitive to pain than others my age. | 2.13 | 1.10 | 2 | 0.67 | -0.33 | 0.04 |
| Q4 | Anxiety increases my sensitivity to pain. | 2.31 | 1.19 | 2 | 0.40 | -0.92 | 0.04 |
| Q5 | I feel stronger pain when injured. | 2.14 | 1.11 | 2 | 0.61 | -0.55 | 0.04 |
| Q6 | Small injuries cause prolonged pain for me. | 1.97 | 1.01 | 2 | 0.82 | -0.08 | 0.03 |
| Q7 | I feel pain more easily due to fatigue than others. | 1.99 | 1.02 | 2 | 0.75 | -0.20 | 0.04 |
| Q8 | My pain experiences are more frequent than most people. | 1.98 | 1.04 | 2 | 0.84 | -0.07 | 0.04 |
| Q9 | When I feel pain, I try to ignore it. | 2.80 | 1.24 | 3 | -0.05 | -1.01 | 0.04 |
| Q10 | I avoid activities that may cause pain. | 3.08 | 1.32 | 3 | -0.29 | -1.06 | 0.05 |
| Q11 | I tell people around me when I feel pain. | 3.39 | 1.22 | 4 | -0.64 | -0.50 | 0.04 |
| Q12 | I can tolerate minor pain without medication. | 3.23 | 1.27 | 3 | -0.42 | -0.85 | 0.04 |
| Q13 | Discussing my pain experience makes me feel better. | 3.18 | 1.23 | 3 | -0.38 | -0.75 | 0.04 |
| Q14 | Accepting pain is part of personal growth. | 3.31 | 1.20 | 4 | -0.56 | -0.50 | 0.04 |
| Q15 | I often expect pain to disappear quickly. | 3.59 | 1.20 | 4 | -0.79 | -0.13 | 0.04 |
| Q16 | Pain is a normal part of everyday life. | 3.18 | 1.28 | 3 | -0.41 | -0.9 | 0.04 |
| Q17 | Pain helps us understand ourselves better. | 2.86 | 1.22 | 3 | -0.08 | -0.91 | 0.04 |
| Q18 | People often exaggerate their pain experiences. | 2.99 | 1.20 | 3 | -0.23 | -0.73 | 0.04 |
| Q19 | Sharing pain is a burden. | 2.43 | 1.13 | 2 | 0.29 | -0.69 | 0.04 |
| Q20 | Pain is a personal experience that should not be shared. | 2.40 | 1.09 | 2 | 0.36 | -0.46 | 0.04 |
| Q21 | Pain is a reminder to slow down. | 3.32 | 1.14 | 3 | -0.56 | -0.29 | 0.04 |
| Q22 | I want others to understand and respect my pain. | 3.62 | 1.14 | 4 | -0.89 | 0.25 | 0.04 |
| Q23 | Pain makes one stronger. | 3.33 | 1.20 | 4 | -0.57 | -0.48 | 0.04 |
| Q24 | My friends and family often ask about my pain. | 3.27 | 1.21 | 3 | -0.42 | -0.65 | 0.04 |
| Q25 | Pain often affects my social life. | 2.39 | 1.12 | 2 | 0.41 | -0.56 | 0.04 |
| Q26 | I hide my pain at school. | 2.64 | 1.18 | 3 | 0.14 | -0.86 | 0.04 |
| Q27 | I have friends at school with whom I share pain. | 3.10 | 1.20 | 3 | -0.38 | -0.77 | 0.04 |
| Q28 | Pain deserves more attention at school. | 2.64 | 1.18 | 3 | 0.09 | -0.85 | 0.04 |
| Q29 | I forget pain more easily when I am with friends. | 3.46 | 1.17 | 4 | -0.69 | -0.23 | 0.04 |

**Appendix D: The Original and Refined Scale Structures**

**
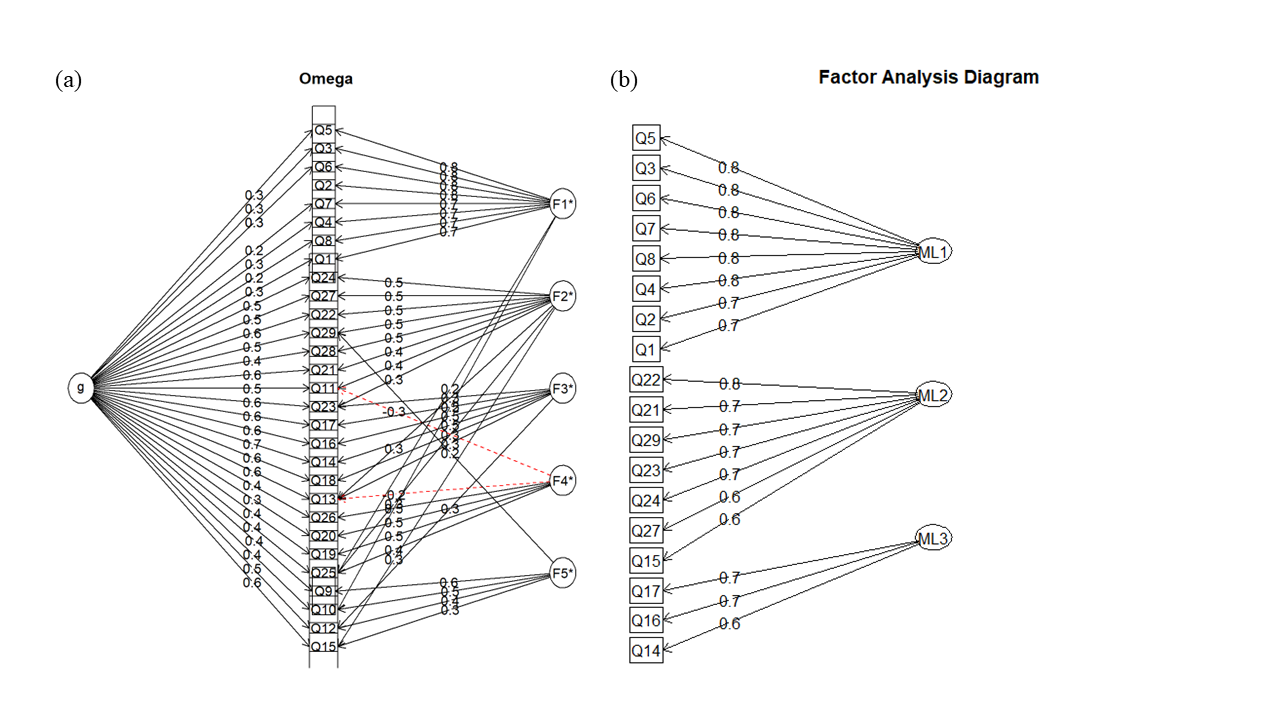
**

**(a)** The initial scale prior to item selection, featuring all observed variables linked to their respective latent factors. The omega model structure is illustrated, indicating factor loadings and inter-item connections. Items with low factor loadings or high cross-loadings (highlighted with red dashed lines) were excluded. **(b)** The optimized factor structure following exploratory and confirmatory factor analyses. Only items with strong factor loadings (>0.6) were retained, resulting in a more streamlined and theoretically sound model. The final structure consisted of three latent factors (ML1, ML2, and ML3), with clearly defined relationships among the retained items.

**Appendix E:**

| **Appendix E: Mean Scores for Each Cluster Across Questionnaire Items** | | | | |
| --- | --- | --- | --- | --- |
| **Variable** | **Question** | **Cluster 1 Mean** | **Cluster 2 Mean** | **Cluster 3 Mean** |
| Q1 | I easily feel pain. | 1.65 | 1.84 | 3.19 |
| Q2 | Small pain is hard to bear. | 1.49 | 1.46 | 2.92 |
| Q3 | I am more sensitive to pain than others my age. | 1.33 | 1.50 | 3.04 |
| Q4 | Anxiety increases my sensitivity to pain. | 1.43 | 1.64 | 3.29 |
| Q5 | I feel stronger pain when injured. | 1.26 | 1.48 | 3.11 |
| Q6 | Small injuries cause prolonged pain for me. | 1.30 | 1.40 | 2.77 |
| Q7 | I feel pain more easily due to fatigue than others. | 1.39 | 1.37 | 2.83 |
| Q8 | My pain experiences are more frequent than most people. | 1.28 | 1.41 | 2.79 |
| Q14 | Accepting pain is part of personal growth. | 1.61 | 3.83 | 3.32 |
| Q15 | I often expect pain to disappear quickly. | 1.82 | 3.98 | 3.76 |
| Q16 | Pain is a normal part of everyday life. | 1.54 | 3.57 | 3.31 |
| Q17 | Pain helps us understand ourselves better. | 1.32 | 3.16 | 3.05 |
| Q21 | Pain is a reminder to slow down. | 1.72 | 3.72 | 3.42 |
| Q22 | I want others to understand and respect my pain. | 1.91 | 4.07 | 3.71 |
| Q23 | Pain makes one stronger. | 1.46 | 3.89 | 3.36 |
| Q24 | My friends and family often ask about my pain. | 1.89 | 3.69 | 3.28 |
| Q27 | I have friends at school with whom I share pain. | 1.51 | 3.38 | 3.32 |
| Q29 | I forget pain more easily when I am with friends. | 1.96 | 3.88 | 3.51 |

**Appendix F:**

| **Appendix F: Standardized Factor Loadings and Path Coefficients in the Structural Equation Model** | | | | | | | |
| --- | --- | --- | --- | --- | --- | --- | --- |
| Indicator | Factor | Estimate | Std. Error | z-value | P(>\|z\|) | Std.lv | Std.all |
| Q1 | Factor1 | 1.000 | - | - | - | 0.482 | 0.667 |
| Q2 | Factor1 | 1.001 | 0.057 | 17.572 | 0 | 0.483 | 0.680 |
| Q3 | Factor1 | 1.115 | 0.056 | 19.928 | 0 | 0.537 | 0.788 |
| Q4 | Factor1 | 1.147 | 0.062 | 18.507 | 0 | 0.553 | 0.722 |
| Q5 | Factor1 | 1.167 | 0.058 | 20.103 | 0 | 0.562 | 0.796 |
| Q6 | Factor1 | 1.002 | 0.052 | 19.349 | 0 | 0.483 | 0.761 |
| Q7 | Factor1 | 0.971 | 0.051 | 18.963 | 0 | 0.468 | 0.743 |
| Q8 | Factor1 | 0.956 | 0.052 | 18.469 | 0 | 0.460 | 0.720 |
| Q21 | Factor2 | 1.000 | - | - | - | 0.541 | 0.709 |
| Q22 | Factor2 | 1.001 | 0.051 | 19.797 | 0 | 0.542 | 0.756 |
| Q23 | Factor2 | 1.121 | 0.056 | 20.013 | 0 | 0.606 | 0.765 |
| Q24 | Factor2 | 0.906 | 0.056 | 16.162 | 0 | 0.490 | 0.609 |
| Q27 | Factor2 | 0.867 | 0.057 | 15.227 | 0 | 0.469 | 0.573 |
| Q29 | Factor2 | 0.933 | 0.054 | 17.416 | 0 | 0.505 | 0.659 |
| Q14 | Factor3 | 1.000 | - | - | - | 0.613 | 0.775 |
| Q15 | Factor3 | 0.768 | 0.044 | 17.377 | 0 | 0.471 | 0.630 |
| Q16 | Factor3 | 0.997 | 0.050 | 20.098 | 0 | 0.611 | 0.725 |
| Q17 | Factor3 | 0.980 | 0.048 | 20.528 | 0 | 0.601 | 0.740 |
| Outcome | Predictor | Estimate | Std. Error | z-value | P(>\|z\|) | Std.lv | Std.all |
| Factor1 | Factor2 | -0.035 | 0.075 | -0.468 | 0.640 | -0.040 | -0.04 |
| Factor1 | Factor3 | 0.126 | 0.067 | 1.864 | 0.062 | 0.160 | 0.160 |
| Factor2 | Factor3 | 0.710 | 0.042 | 16.797 | 0 | 0.805 | 0.805 |

**Appendix G: Sample Survey Items**

The Pain Sensitivity and Coping Strategies Questionnaire (APSIA) is designed to assess adolescents' perceptions of pain and their coping mechanisms. Please respond to each statement based on your personal experiences, using the following scale:

1. Strongly Disagree
2. Disagree
3. Neutral
4. Agree
5. Strongly Agree

**Pain Sensitivity and Coping Strategies Questionnaire (APSIA)**

| **Item** | **Statement** | **1** | **2** | **3** | **4** | **5** |
| --- | --- | --- | --- | --- | --- | --- |
| Q1 | I easily feel pain. |  |  |  |  |  |
| Q2 | Small pain is hard to bear. |  |  |  |  |  |
| Q3 | I am more sensitive to pain than others my age. |  |  |  |  |  |
| Q4 | Anxiety increases my sensitivity to pain. |  |  |  |  |  |
| Q5 | I feel stronger pain than other people when injured. |  |  |  |  |  |
| Q6 | Small injuries cause prolonged pain for me. |  |  |  |  |  |
| Q7 | I feel pain more easily due to fatigue than others. |  |  |  |  |  |
| Q8 | My pain experiences are more frequent than most people. |  |  |  |  |  |
| Q9 | Pain is a reminder to slow down. |  |  |  |  |  |
| Q10 | I want others to understand and respect my pain. |  |  |  |  |  |
| Q11 | Pain makes one stronger. |  |  |  |  |  |
| Q12 | My friends and family often ask about my pain. |  |  |  |  |  |
| Q13 | I have friends at school with whom I share pain. |  |  |  |  |  |
| Q14 | I forget pain more easily when I am with friends. |  |  |  |  |  |
| Q15 | Accepting pain is part of personal growth. |  |  |  |  |  |
| Q16 | I often expect pain to disappear quickly. |  |  |  |  |  |
| Q17 | Pain is a normal part of everyday life. |  |  |  |  |  |
| Q18 | Pain helps us understand ourselves better. |  |  |  |  |  |

Please indicate your level of agreement with each statement by marking the appropriate column. Your responses will help us understand your pain perception and coping strategies.
